# Colecionismo e contextos coloniais no Museu Nacional de Arqueologia e Museu Municipal Santos Rocha, 1893-1930

**DOI:** 10.1590/S0104-59702025000100043

**Published:** 2025-09-29

**Authors:** Elisabete Pereira, Liliana Caldeira, Maria Figueira, Francisca Laevski, Ana Margarida Ferreira, Quintino Lopes

**Affiliations:** i Investigadora, IN2PAST/Instituto de História Contemporânea/Universidade de Évora. Évora – Portugal. ejsp@uevora.pt; ii Doutoranda em História, Universidade de Évora. Évora – Portugal. liliana.caldeira@hotmail.com; iii Bolseira, Projeto TRANSMAT/Museu Municipal Santos Rocha. Figueira da Foz – Portugal; iv Doutoranda, IN2PAST/Instituto de História Contemporânea/Universidade de Évora. Évora – Portugal. figueimaria@gmail.com; v Mestre em Ensino de História; Faculdade de Psicologia/Universidade de Lisboa. Lisboa – Portugal. franciscalaevski@gmail.com; vi Conservadora, Museu Municipal Santos Rocha. Figueira da Foz – Portugal. ana.ferreira@cm-figfoz.pt; vii Investigador, IN2PAST/Instituto de História Contemporânea/Universidade de Évora. Évora – Portugal. qmjl@uevora.pt

**Keywords:** Museu Nacional de Arqueologia, Museu Municipal Santos Rocha, História de coleções, História colonial, Pesquisa de proveniência, Museu Nacional de Arqueologia, Museu Municipal Santos Rocha, History of collections, Colonial history, Provenance research

## Abstract

Mais de dois mil objetos transnacionais estão hoje no Museu Nacional de Arqueologia, em Lisboa, e no Museu Municipal Santos Rocha, na Figueira da Foz. Sua história está relacionada com o nacionalismo, o colonialismo e o desenvolvimento da arqueologia pré-histórica no final do século XIX e início do século XX. Cruzando múltiplas fontes e publicações de época, o artigo aborda o itinerário dessas coleções decorrentes, principalmente, das campanhas militares de ocupação de territórios africanos e das redes de poder que transportaram até Portugal múltiplos artefactos. As narrativas, descrições e categorizações eurocêntricas que perduram desde essa época mostram como a pesquisa de proveniência é fundamental para documentar e confrontar os legados coloniais dos museus.

## Abstract (en)

Over two thousand transnational objects (most with colonial origins) are currently in the Museu Nacional de Arqueologia in Lisbon and the Museu Municipal Santos Rocha in Figueira da Foz. Their histories are linked to nationalism, colonialism and the development of prehistoric archaeology in the late nineteenth and early twentieth centuries. This article cross-references multiple sources and periodicals to trace the journeys of these collections (primarily involving military campaigns in Africa) and the power networks that brought these objects to Portugal. The Eurocentric narratives, descriptions and categorizations that have endured since that time show how provenance research is essential to document and confront the colonial legacies of museums.

O estudo de coleções confundiu-se ao longo do tempo com uma crónica de coleções, dado que uma das primeiras tarefas dos conservadores e gestores de museus consistia na elaboração de um inventário ou catálogo, uma exigência institucional, académica e mesmo jurídica (Poulot, 2021, p.38). Apesar de existirem essas crónicas e/ou inventários de coleções, e mais recentemente os catálogos digitais *online*, a maioria dos museus portugueses, quando inquiridos sobre coleções específicas, como as de proveniência não europeia, não possui respostas concretas relativamente a composição e dimensão, historial de objetos, contextos de proveniência, recolha ou de incorporação nos museus (Amaro, Felismino, 2021). Esse fato prende-se com a dificuldade da maioria das instituições museológicas em promover internamente investigação sobre as suas coleções, frequentemente por insuficiência de recursos humanos. A investigação desenvolvida pelas equipas dos museus em cooperação com projetos académicos pode contribuir para o enriquecimento da documentação das coleções, fomentando novas narrativas. O projeto de investigação “TRANSMAT – Materialidades transnacionais: reconstituir colecções e conectar histórias (1850-1930)”^1^ procurou precisamente, por meio do trabalho colaborativo, contribuir para um conhecimento mais detalhado das coleções transnacionais de dois museus portugueses (Pereira, Carvalho, Cardoso, 2021).

Neste artigo abordamos as coleções designadas, no final do século XIX e início do século XX, como “comparativas” nos museus de arqueologia, sendo o nosso *focus* transnacional relacionado com os objetos não europeus, que surgem nesses museus por exigência do desenvolvimento da pré-história como disciplina, a partir da segunda metade do século XIX. Essa perspetiva englobava a necessidade de recolha de objetos arqueológicos e artefactos contemporâneos não europeus, qualificados como “etnográficos” e utilizados para comparar o desenvolvimento tecnológico e os contextos culturais (Quiblier, 2014; Podgorny, 1999).

Desde o século XVIII, o mesmo tipo de artefactos era utilizado para o desenvolvimento dos estudos de antropologia e etnografia, sendo essa tipologia de coleções integrada, numa fase inicial, em seções específicas nos museus de história natural. As culturas não europeias eram entendidas não como pertencentes a um presente distinto, mas como uma fase histórica ultrapassada. Os museus e as coleções etnográficos das metrópoles imperiais exibiam, então, por meio de “objetos” o seu poder sobre as culturas colonizadas. Nas secções comparativas dos museus de arqueologia, a mesma tipologia de artefactos começou a ser utilizada como instrumento de compreensão do passado remoto (Schnapp, 2020; Oleaga, Monge, 2009).

Acompanhando o movimento científico internacional, em Portugal as coleções de artefactos não europeus começaram por surgir nos gabinetes de curiosidades a partir do século XVI (Brigola, 2019) e, no século XVIII, persistiam em várias coleções privadas e na Academia das Ciências de Lisboa. Objetos também provenientes de contextos não europeus foram incorporados no Museu de Etnografia da Universidade de Coimbra, que surgiu em 1881, e na mesma década na Sociedade de Geografia de Lisboa, cujo Museu de História e Etnografia foi criado em 1884. A Faculdade de Ciências da Universidade do Porto possuía a mesma tipologia de coleções no seu Museu e Laboratório, criado em 1911. Em 1965 foi criado o Museu de Etnologia do Ultramar, que, em 1973, será renomeado como Museu de Etnologia. As coleções não europeias existiam desse modo nos museus etnográficos das instituições mencionadas, mas também em diversos museus com coleções de arqueologia, especialmente pré-histórica, com fins comparativos.

Essa integração das coleções etnográficas não europeias nos museus de arqueologia em Portugal não foi até agora estudada, do ponto de vista histórico, na sua relação com a institucionalização da disciplina nem nas suas relações com o colonialismo. A questão tem sido, aliás, um tema insuficientemente abordado na historiografia.^2^ Este artigo contribuirá para essa linha de investigação, procurando identificar esses objetos transnacionais em dois museus portugueses, reconstituir as suas proveniências, os seus contextos de aquisição, a época de incorporação, os seus significados e itinerários antes de chegarem aos museus e depois de integrarem as coleções.

Para este estudo, recorremos em primeiro lugar à burocracia institucional. Os catálogos dos museus, digitalizados ou em papel, são, como mostrou Hannah Turner (2020), objetos com a sua própria história e complexidade. Essa densidade manifesta-se quando, ao tentarmos reconstituir as histórias dos artefactos, nos deparamos com a necessidade de compreender e reconstituir os processos de registo, as pessoas envolvidas nesse trabalho, as suas referências e fontes de informação, as redes que ligavam indivíduos em vastos territórios e canalizavam os artefactos para os museus. A produção de “objetos etnográficos” é, como menciona Turner (2020, p.186), simultaneamente produção de conhecimento e de identidade: sobre a própria instituição que promove as coleções, sobre os atores associados aos processos de registo, sobre as comunidades cujo património cultural é definido pelo *staff* do museu a milhares de quilómetros de distância, com informação indireta e descontextualizada. Os arquivos das duas instituições museológicas com que trabalhámos não são repositórios neutros de informações, mas instrumentos ativos de produção de conhecimento sobre a história colonial. Os registos produzidos no século XIX e início do século XX serviram para legitimar o domínio colonial ao transformar informação parcial ou enviesada em evidências “confiáveis” que foram sendo reproduzidas ao longo dos anos.

Essa burocracia institucional, que está na base do desenvolvimento da ciência e do conhecimento (Podgorny, 2021), constitui uma fonte histórica basilar para o desenvolvimento do estudo dessas coleções comparativas, permitindo efetivar a pesquisa de proveniência (Deußen, Karakis, 2022) e reconstituição dos itinerários de objetos (Appadurai, 1986; Alberti, 2005; Pereira, Nunes, Lopes, 2019). Centramos a nossa investigação, neste artigo, na análise das coleções “comparativas” do Museu Nacional de Arqueologia (MNA) e do Museu Municipal Santos Rocha (MMSR) desde o momento da sua criação e durante o período em que assumem protagonismo no projeto científico das duas instituições. No caso do museu de Lisboa, consideramos que isso ocorre durante o período de direção, entre 1893 e 1929, do seu fundador e primeiro diretor, José Leite de Vasconcelos (1858-1941). No caso do museu da Figueira da Foz, analisamos o período correspondente à direção de António dos Santos Rocha (1853-1910), igualmente fundador e primeiro diretor, entre 1893 e 1910.^3^

Reconstituindo a biografia dos doadores e de uma parte dos objetos, procurámos entender os contextos de recolha e de doação, bem como a sua relação com os contextos administrativos e militares coloniais. Para tal, foi necessário a consulta de literatura produzida durante o período em causa e fontes de diversos arquivos nacionais, cruzando com a informação de arquivos locais e dos próprios museus. A análise dos dados, adaptando um modelo de estudo de coleções desenvolvido por Marta Lourenço e Samuel Guessner (2014), permitiu desenvolver a investigação que apresentamos neste artigo e permitirá, no futuro, aprofundar diversos aspetos relacionados com a pesquisa de proveniência de coleções e as biografias de objetos. Tal como no modelo de Guessner e Lourenço, a nossa base de dados considera vários campos de análise das coleções e dos objetos: em primeiro lugar, uma distinção entre o objeto/instrumento individual – por exemplo, um amuleto ou um instrumento musical – e a classe de objetos/instrumentos que partilham a mesma designação; em segundo lugar, uma distinção entre aspetos sincrónicos, resultantes da inspeção direta dos objetos,^4^ e aspetos diacrónicos relacionados com a sua história. Como salientam os autores citados, as questões formuladas durante o estudo dos objetos e das coleções são mutuamente dependentes e igualmente importantes para a sua compreensão, mas, como são de natureza diferente, dependem de diferentes tipos de fontes e exigem diferentes métodos para a sua resolução. Essa sistematização constituiu a base da pesquisa de proveniência (Bachmann, Berazategui, 2022; Ebeling, 2022) que apresentamos neste artigo relativamente a alguns objetos e coleções.

## Coleções “etnográficas” nos museus de arqueologia

Durante a segunda metade do século XIX, colecionadores e cientistas começaram a recolher nas coleções particulares ou nos museus os testemunhos dessas sociedades ditas “primitivas” como resultado da aceitação das perspetivas evolucionistas e da origem pré-diluviana da humanidade (Geroulanos, 2024, p.57-61). Do ponto de vista da encenação, esses artefactos eram exibidos em seções específicas, com estratégias e tecnologias de exposição adequadas.

Na Suíça, o Museu de Antiguidades de Lausanne, criado em 1852, exibia uma coleção etnográfica com o objetivo de evidenciar os “vários graus de desenvolvimento pelos quais os povos passam antes de alcançar a civilização propriamente dita” (Troyon, 1858, p.2). No relatório produzido em 1858 identificam-se três seções: “Antiguidades Nacionais”, “Antiguidades estrangeiras na Suíça” e “Coleção de Etnologia”. Esta última era consagrada à “exposição de produtos da indústria moderna entre povos estrangeiros à civilização moderna” (Troyon, 1858, p.12) e exibia objetos da América do Norte, das Antilhas, da América do Sul, da Ásia e de África. Em França, no Museu Nacional de Arqueologia de Saint-Germain-en-Laye, as coleções etnográficas dos povos à época designados como “selvagens contemporâneos”, bem como as coleções arqueológicas estrangeiras, eram exibidas em 65 vitrines na “grande sala de comparação” (Reinach, 1887, p.75-102), que exibia objetos provenientes de vários pontos da Europa, da Ásia, da América, de África e da Austrália. Essa sala deveria ser, de acordo com as instruções de Salomon Reinach, o corolário da visita a todo o museu, ou seja, das anteriores 28 salas, de forma a ser possível ao visitante a perceção da sua identidade, nesse caso a francesa, em relação à identidade dos outros povos (Estoile, 2010, p.12). Enquanto no Museu Nacional em Saint-Germain-en-Laye essas coleções serviam como contraste com a arqueologia nacional, no Museo Canario (Canary Museum) criado em Las Palmas, Grande Canária, em 1879 (Girón Sierra, Betancor Gómez, 2023), ou no Museu de Pré-História de Eyzes-de-Tayac, criado em 1918, em França, os artefactos não europeus auxiliavam a compreensão de uma pré-história regional. Bertrand Daugeron (2009, p.147) refere que “o projeto comparativo anima todos os primeiros museus arqueológicos europeus do século XIX”. Contudo, como reflete a história do museu de Pré-História de Eyzies-de-Tayac, essa tendência estende-se ao século XX.

Em Portugal encontramos essa mesma propensão. Veja-se a justificação de José Leite de Vasconcelos, diretor e fundador do MNA, em 1915: “A coleção que organizei no Museu Etnológico tem por fim sobretudo … pôr diante dos olhos dos que visitam a seção pré-histórica exemplares etnográficos dos selvagens que ajudem a entender o modo de viver e a arte dos homens primitivos” (Vasconcelos, 1915, p.261). Tal como no museu regional de Eizies-de-Tayac, na Figueira da Foz, no centro de Portugal, António dos Santos Rocha registava igualmente a existência de uma coleção comparativa no museu que dirigia: “*2.ª Seção Etnográfica dos povos selvagens dos tempos modernos*” (Rocha, 1905, p.10). Santos Rocha e Vasconcelos acompanhavam e adaptavam ao contexto português as práticas dos já referenciados Musée d’Archeologie National, Museu de Antiguidades de Lausanne, Museu de Pré-História de Eyzes-de-Tayac, mas também do Museu Pitt Rivers (Morton, 2012) ou do Museu Arqueológico e Etnográfico de Modena (Falcucci, 2022), onde as coleções comparativas eram igualmente uma realidade.

**Figura 1:**
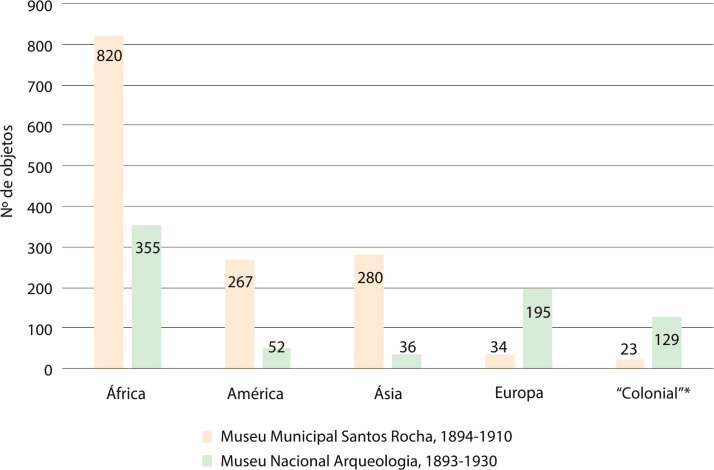
: Gráfico de análise de proveniências da coleção comparativa do MNA e MMSR (Fonte: O Arqueólogo Português*,* 1895-1920; Livro de Entradas*,* 1906-1930; Museu Municipal da Figueira, 1893a,1893b; Rocha, 1905, 1907a, 1907b, 1909, [1910])

Objetos pré-históricos, romanos ou contemporâneos, provenientes de Espanha, França ou Itália, constavam nas coleções do museu de Lisboa. A cultura material do Egito antigo, por exemplo, tem presença generalizada e bem estabelecida nos museus de todo o mundo (Stevenson, 2019), e não era exceção no MNA. Nesse âmbito transnacional, África constituía o continente mais bem representado no MNA (ver Figura 1). A proveniência africana era igualmente prevalecente nas coleções transnacionais do Museu da Figueira da Foz. Existiam objetos provenientes de Espanha, de Itália, também da Rússia, Suíça, Turquia, dos continentes americano – onde se destacam objetos trazidos do Brasil – e asiático, mas, como reflete o gráfico na Figura 1, predominavam igualmente os objetos provenientes do continente africano.

*A coluna “colonial” corresponde a objetos sem proveniência precisa ou registada como proveniente do império colonial português.

Recorde-se que ambas as instituições foram criadas na última década do século XIX, em 1893 e 1894, numa época de reforço de ocupação efetiva dos territórios africanos, consequência da Conferência de Berlim (1884-1885). A então denominada “África Portuguesa” abrangia Guiné-Bissau, Angola, São Tomé e Príncipe, Cabo Verde e Moçambique. Depois de 1885, consequência da implementação do princípio de *uti possidetis jure* (ou seja, apenas os que de fato ocupam um território possuem direito sobre este), que rejeitava reconhecer os “direitos históricos” reclamados pelos portugueses sobre os territórios, o país desenvolveu ações militares efetivas para eliminar as resistências das populações à sua administração e exploração territorial.

## A coleção comparativa do Museu Nacional de Arqueologia

Apesar de identificarmos objetos de proveniência transnacional desde o primeiro ano de vida do museu, criado em dezembro de 1893 (Vasconcelos, 1895, p.218), a coleção comparativa do MNA^5^ terá tomado forma sobretudo a partir de 1904, quando é doada uma “coleção de armas, utensilios e instrumentos musicos [*sic*] dos indígenas do Ibo” (Nigéria) (Vasconcelos, 1904, p.310). Seguem-se diversas doações em 1905 e 1906, e as compras do próprio diretor, também em 1906, de dezenas de artefactos, sobretudo armas, registados como provenientes da “África Portuguesa”, da Índia, de Angola e de São Tomé (Campos, 1907, p.111). Em 1915 a instituição encontrava-se dividida em seis seções, sendo a quarta dedicada à comparação e denominada “Seção comparativa”.^6^ Essa seção encontrava-se por sua vez subdividida em quatro coleções: “objetos coloniais; objetos estrangeiros antigos e modernos; objetos vários (estante móvel com desenhos); objetos modernos que servem para explicar o passado” (Vasconcelos, 1915, p.261). Os mencionados “objetos coloniais” provinham, como vimos, em grande medida de África, mas também de Timor e da Índia, como especifica o diretor (p.261-262).

Na ausência de um livro de registo de entradas, criado em 1906, mais de uma década depois da fundação do museu, é nos volumes da revista *O Arqueólogo Português* que se identificam mais de duas centenas de registos de incorporação de objetos avulsos ou conjuntos de objetos de origem transnacional. Esses registos surgiam no âmbito dos artigos publicados sob a designação “Aquisições do Museu Etnográfico Português” e “Aquisições do Museu Etnológico Português”. O termo “aquisições” comportava a incorporação de objetos por meio de doações, de trocas, de compras a particulares, a casas comerciais ou em leilões. Apesar de ter sido criado o *Livro de Entradas* em 1906, as incorporações continuaram a ser divulgadas n’*O Arqueólogo Português* até 1919-1920.^7^ A nossa análise comporta, desse modo, a descrição das aquisições de objetos de cariz transnacional publicadas na revista do MNA entre 1895^8^ e 1919-1920, em confronto com os registos do *Livro de Entradas*, entre 1906 e 1930. Publicados exclusivamente na primeira série da mencionada revista, devemos sublinhar o fato de coexistirem formatos imprecisos de registo, o que condicionou a investigação quantitativa da coleção, uma vez que não foi possível confrontar os registos com os objetos existentes nas reservas da instituição museológica. Apesar de predominarem as incorporações de objetos descritos individualmente (por exemplo, “panela [‘cocron’] dos Caingangues de Goio-Chê, ou de Água-Preta”, em Vasconcelos, 1913, p.167), encontramos registos indefinidos de incorporação de coleções (por exemplo, “70 armas dos indigenas da África Portuguesa: azagaias, lanças, punhais, espadas, travesseiros e um tambor”, em Campos, 1907, p.107) e coleções de objetos não quantificados (por exemplo, “ofereceu várias lascas de silex”, em Campos, 1907, p.106). Esses formatos tornam imprecisa a identificação de objetos específicos, mas não impedem o conhecimento da parte quantificável. Deve salientar-se que foram registados na revista *O Arqueólogo Português* e no *Livro de Entradas* a incorporação de dez coleções de objetos não quantificáveis, assim como o registo individual de 768 objetos arqueológicos e etnográficos^9^ de diversas proveniências, doados por 66 atores, incluindo atores institucionais. Para o período em análise, José Leite de Vasconcelos surge como o principal protagonista, tendo sido responsável pela incorporação de 349 dos objetos transnacionais registados. Os registos permitem verificar que a maioria da coleção foi comprada pelo próprio museu por meio de Vasconcelos.^10^

Do conjunto de maiores doadores salienta-se igualmente César Azevedo Pires (?-1936), farmacêutico em Lisboa (Carvalhaes, 1908, p.63), que doou entre 1904 e 1908 (Campos, 1907, p.224-225) 64 objetos provenientes de África (“Angola”, “Moçambique”, “Zambézia”) e cinco objetos provenientes da Gruta de Altamira (Espanha) (Vasconcelos, 1905, p.47). César Azevedo Pires desenvolveu investigação nas áreas da pré-história e da proto-história, foi sócio da Associação dos Arqueólogos Portugueses (Vasconcelos, 1905, p.47) e da Sociedade Farmacêutica Lusitana,^11^ publicando os seus trabalhos em várias revistas e jornais (César..., c.1950, p.937). Tal como se verifica relativamente a José Leite de Vasconcelos, não se identificou qualquer deslocação sua aos territórios africanos.

O mesmo não acontece com Alfredo Freire de Andrade (1859-1929), diretor-geral das colónias entre 1911 e 1914, que ofereceu em 1912 “oito instrumentos de pedra lascada”, provenientes de Mailana (Moçambique) (Livro de Entradas, 1906-1930, fl.61). Também o capitão Oliveira e Santos, governador do distrito da Lunda (Angola) e responsável pelo plano de subordinação dos “Quiocos” (tchokwe, etnia banta; povo que habita o sul da República Democrática do Congo, o nordeste de Angola e o noroeste da Zâmbia), em 1920 (Pélissier, 2013, p.389-390), ofereceu um “machado de ferro”, uma máscara “muquiche” e um “feitiço”, todos da Lunda (Angola) (Livro de Entradas, 1906-1930, fl.95). Nessa região decorreram as campanhas militares contra o soba Bunla-Bunla e os Bondos, em finais de 1916 e inícios de 1918. No ano seguinte existiram campanhas militares contra o soba Gunza (Pélissier, 2013, p.330). Estamos assim perante doadores que obtiveram os objetos no contexto de uma posição de poder sobre os povos africanos, embora no caso de Freire de Andrade se trate de objetos provenientes de sítios arqueológicos, e não de objetos saqueados durante campanhas militares.

Também no caso das doações do capitão Carlos Maia Pinto (1866-1932) (Caldeira, 2023, p.61-63) ao MNA, em 1916, três objetos provenientes de Angola foram obtidos no âmbito das campanhas militares que dirigiu. Surgem registados no *Livro de Entradas*: “6882 Máscara de madeira … 6883 ‘Quinda’. De uso doméstico para guardar milho, amendoim etc.; 6884 Cinto-cartucheira com polvorinho dos Cuanhamas”. A doação ficou registada também em carta de Carlos Maia Pinto (14 fev. 1916) a José Leite de Vasconcelos, na qual descreve a doação. Maia Pinto comandou as operações militares no norte de Angola em 1913, coadjuvado por David Magno (1877-1957), que deixou registo sobre o contexto de aquisição dos artefactos: “Não pude evitar que a coluna se apoderasse de um grande despojo de gado e de um verdadeiro arsenal de amuletos e feitiços, espadas, instrumentos bélicos, saias de tiras de coiro e capacetes” (Desde a inauguração..., s.d., p.46).

Desde 1872 que a referida região dos Dembos (norte de Angola) se manifestava contra a autoridade portuguesa. De 1872 até 1919 foram organizadas contra os Dembos várias expedições militares até que “a impossibilidade de arranjar pólvora, a doença do sono, a abertura de estradas e a cultura do café iriam anular os últimos lutadores pela independência pelo menos até 1961” (Marracho, 2008, p.iv). Os objetos oferecidos por Carlos Maia Pinto materializam, como se verifica, a grande resistência das populações africanas e a violência da efetiva ocupação dos territórios.

**Figura 2:**
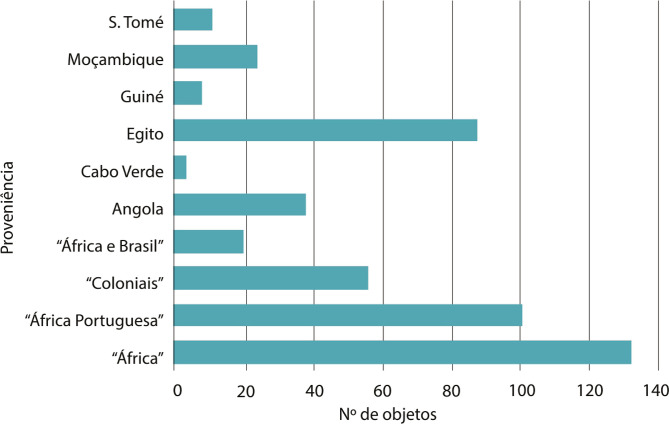
: Gráfico de objetos do Museu Nacional de Arqueologia provenientes do continente africano (Fonte: O Arqueólogo Português, 1893-1920; Livro de Entradas, 1906-1930)

No que respeita à proveniência, essa coleção é constituída maioritariamente por objetos oriundos do continente africano. Contudo, a informação sobre as procedências geográficas são na sua maioria muito vagas, o que revela a desconsideração sobre as comunidades e culturas locais de origem no projeto científico dos museus de arqueologia. Numa parte considerável encontramos apenas a indicação de “África Portuguesa” ou “África”. Noutros casos as doações têm o registo “África e Brasil”, “África Oriental” ou foram simplesmente registadas como de proveniência “colonial”. Nos casos em que a proveniência é mais precisa, é possível verificar um maior número de registos relativos ao Egito,^12^ a Angola, a Moçambique e a São Tomé (ver Figura 2).

**Figura 3:**
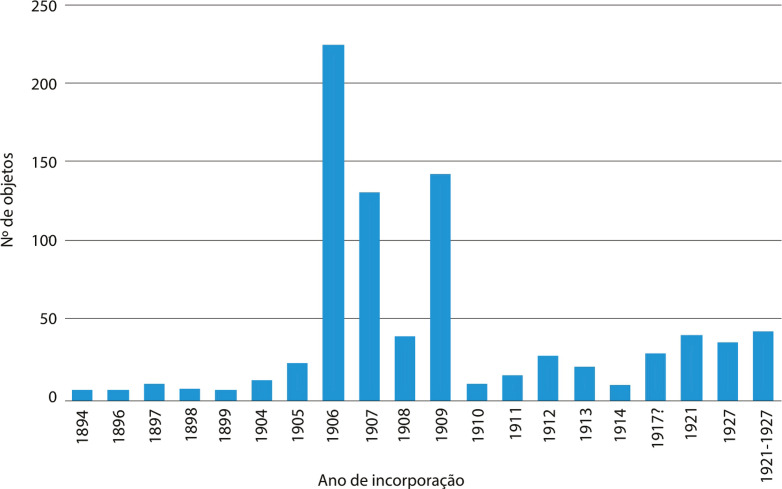
: Gráfico da cronologia da incorporação de objetos transnacionais no Museu Nacional de Arqueologia (Fonte: O Arqueólogo Português, 1893-1920; Livro de Entradas, 1906-1930)

Se atentarmos ao movimento cronológico de entrada dos objetos, verificamos que a maior quantidade foi integrada nas coleções do MNA entre 1906 e 1909 (Figura 3), num período de intensas campanhas militares em Angola, Moçambique e Guiné.^13^

No que respeita ao contexto de aquisição, José Leite de Vasconcelos, tal como César Azevedo Pires, farmacêutico de Lisboa, não tinha qualquer ligação direta com os territórios coloniais, mas ambos tiveram acesso a um conjunto considerável de objetos provenientes de África, muito provavelmente via as redes de poder colonial, como os militares e administradores coloniais, que se movimentavam entre a metrópole e os territórios africanos. Parte desses agentes coloniais elaboraram diários e relatórios, e alguns publicaram estudos etnográficos em que registaram as suas interpretações distorcidas e eurocêntricas do modo de vida das populações e também do contexto de recolha dos artefactos, parte deles por meio das expedições militares, como as anteriormente citadas.

**Figura 4:**
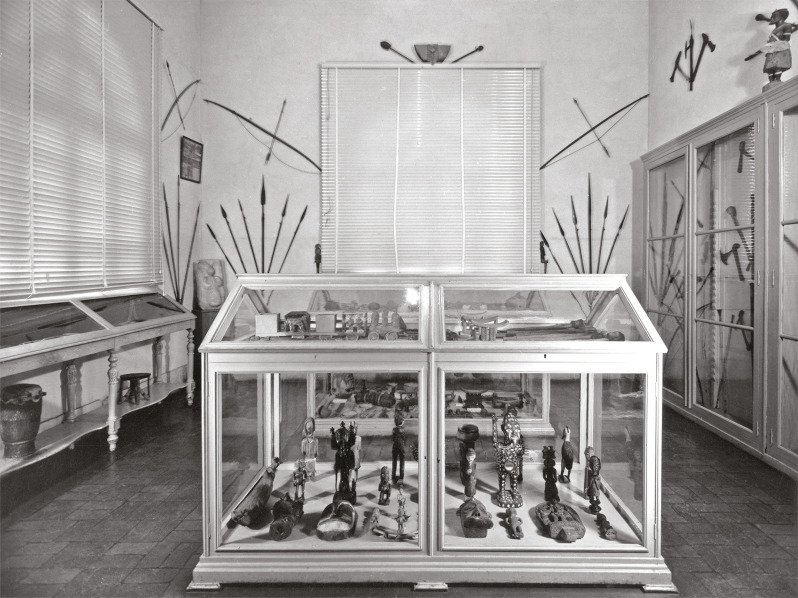
: “Sala de Etnografia Ultramarina” no Museu Nacional de Arqueologia, c.1960 (Fonte: Arquivo do Museu Nacional de Arqueologia)

**Figura 5:**
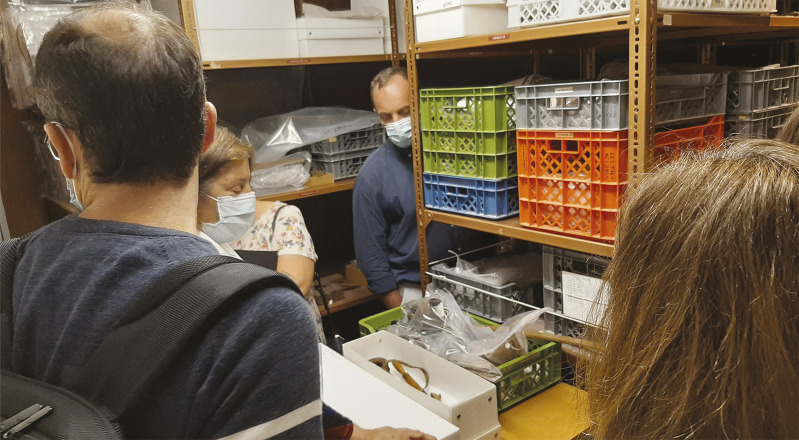
: Visita da equipa do projeto TRANSMAT às reservas do Museu Nacional de Arqueologia, onde se encontra a coleção comparativa, 28 de outubro de 2021 (Fonte: Elisabete Pereira)

A seção comparativa assumiria, com o passar dos anos, novas designações e encenações. O arquivo do Museu Nacional de Arqueologia não tem fotografias ilustrativas da coleção comparativa do período em análise.^14^ Em meados do século XX, a obra de João Saavedra Machado (1965) mostra-nos que a coleção, antes designada “seção colonial”, passou a denominar-se “Sala de Etnografia Ultramarina”, essa sim registada fotograficamente (Figura 4). Um relatório, publicado em 1974-1976, informa que a coleção permaneceu praticamente intacta desde a sua criação, mas, no final da década de 1960, foi intervencionada com o intuito de reduzir a sua exposição para favorecer as “salas de arqueologia estrangeira e egípcia anexas”. A seção colonial foi, então, privada de uma sala, e mais de metade da coleção foi, segundo se descreve no “Relatório sobre a seção colonial do Museu Nacional de Arqueologia e Etnologia”, colocada nas reservas da Seção de Etnografia e Etnografia Portuguesa, e algumas peças ficaram “dispersas por vários gabinetes e lugares do Museu” (Pereira, 1974-1977, p.23). Foi, na década de 1980, retirada de exposição e colocada nas reservas do MNA, onde a encontrámos no início do projeto TRANSMAT, em 2021, acondicionada em várias estantes (Figura 5).

## A coleção comparativa do Museu Municipal Santos Rocha

A denominada “seção de etnografia … que é propriamente de comparação” encontra-se perfeitamente formulada no Regulamento Interno do Museu da Figueira da Foz de 1894 (Ata da Câmara, 26 abr. 1894, f.72v) e de 1905 (Rocha, 1905). Por meio desses documentos pode conhecer-se a sua organização e o lugar da comparação via objetos transnacionais (Rocha, 1905, p.9-10). A criação dessa coleção foi possível devido à colaboração de 59 atores que conduziram para o museu 1.424 objetos doados entre 1893 (ano de formação das primeiras coleções do museu) e 1910. Parte dos atores enunciados eram depositantes que, de acordo com o regulamento da instituição, não deixavam de constituir os legítimos proprietários dos objetos. Para efetivar essa possibilidade e registar o movimento das coleções, António dos Santos Rocha criou o livro de “Registo de Saída dos Depósitos” (p.13-14). Foi efetivamente pela via das doações e depósitos que o Museu adquiriu a maioria dos seus objetos, embora estivesse também registado no regulamento a possibilidade de aquisição por compra ou troca (p.12).

A proposta de criação de um museu na Figueira da Foz data de 1892. Criada uma comissão organizadora, no ano seguinte foi instalado provisoriamente numa parte do edifício designado como Casa do Paço (Rocha, 1905, p.6). No contexto de criação desse primeiro espaço expositivo, produziu-se o mencionado “Registo das Entradas por Depósito” e também “Registo das Entradas por Donativo”. Preparados com minúcia tipográfica, que estabeleceu a grelha de registos manuscritos, contrasta com as práticas de gestão da instituição criada por José Leite de Vasconcelos, em Lisboa, que, ambicionando o desenvolvimento de um museu nacional, recrutou, tal como Adolf Bastien na Alemanha (Kuper, 2023, p.81), dezenas de angariadores de objetos e coleções com o argumento de que reunidos na instituição, os objetos eram preservados e valorizados (Pereira, 2018, p.155-234). Contudo, uma capacidade de registo e organização limitada tornou parte deles impercetíveis até à atualidade, como demonstra o recente estudo sobre a coleção doada por Alberto Osório de Castro ao MNA (Caldeira, 2023, p.80-92).

No MMSR, as coleções foram minuciosamente descritas. Nos livros referidos encontram-se registados, além dos nomes dos doadores e depositantes dos objetos, outras informações, desde breves descrições a informações pormenorizadas sobre a data e o contexto da recolha dos objetos, por vezes os seus usos ou os grupos populacionais a que pertenceram, bem como a identificação de outros atores envolvidos no seu percurso. Foram posteriormente agregados diversos registos, sempre assinados e datados, assinalando, por exemplo, a mudança de seção “para dar lugar a objetos melhores”.

**Figura 6:**
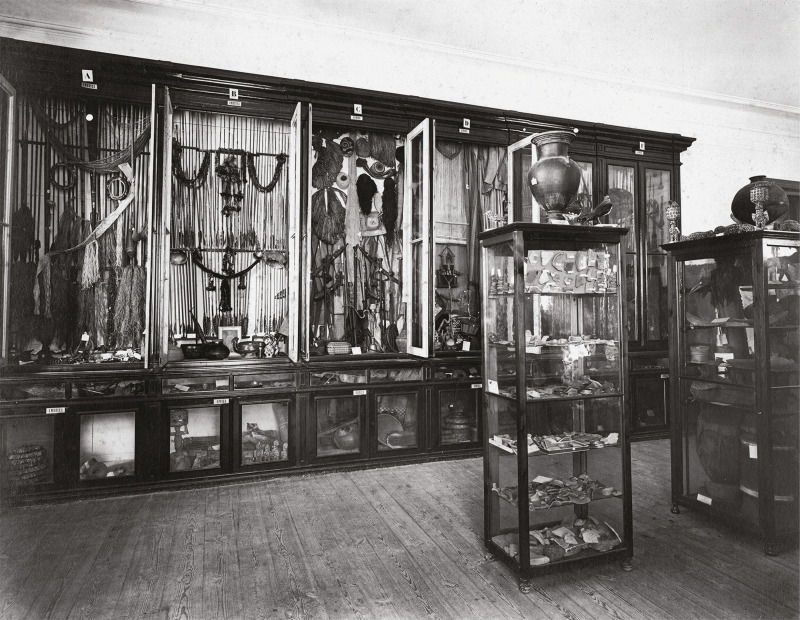
: Sala de comparação, c.1905 (Fonte: Arquivo do Museu Municipal Santos Rocha)

Uma análise do âmbito cronológico de incorporação de objetos transnacionais no MMSR, cuja seção comparativa encontramos documentada em fotografias de época (Figura 6), verificamos, em comparação com o MNA, uma maior diversidade relativamente aos períodos de incorporação, bem como a existência de ofertas anteriores à inauguração oficial do museu que ocorreu, recorde-se, em 1894. O ano anterior, 1893, constitui, no MMSR, o período de incorporação de maior número de objetos comparativos, maioritariamente provenientes de África e América do Sul. É incorporada e noticiada na imprensa local, por exemplo, uma coleção de objetos provenientes do “Congo”, África Central, doados por Antonio d’Oliveira e Silva Júnior (1845-1908) (Museu..., 11 maio 1895). O mesmo ator irá continuar a contribuir para o museu, sendo um dos principais doadores entre 1895 e 1897.

**Figura 7:**
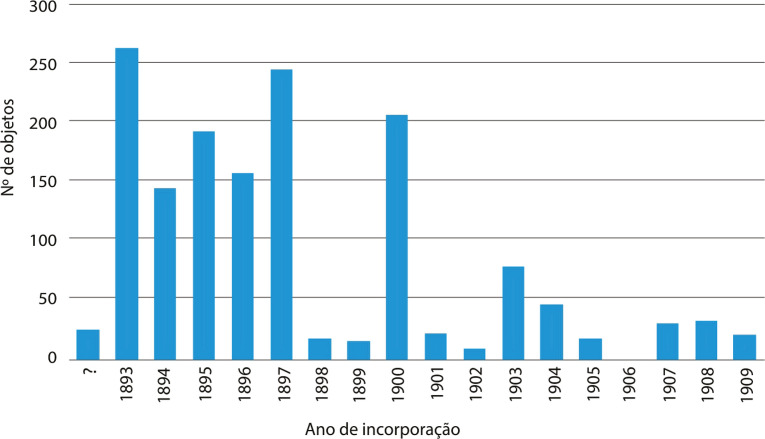
: Gráfico da cronologia da incorporação de objetos transnacionais no Museu Municipal Santos Rocha (Fonte: Museu Municipal da Figueira, 1893a, 1893b; Rocha, 1905, 1907a, 1907b, 1909, [1910])

Natural da Figueira da Foz (Museu Municipal da Figueira, 1893b), Antonio d’Oliveira e Silva Júnior residiu em Luanda e exerceu o cargo de “Chefe do Concelho do Dondo”, em Moçambique,^15^ região de proveniência de alguns dos objetos por ele doados. Outros objetos são provenientes da região dos Dembos, em Angola, um dos povos que, como referimos, mais resistiu à ocupação portuguesa entre 1872 e 1919 (Marracho, 2008; Pélissier, 2013). Também em 1893, Bernardo Augusto Lopes (1821-1916), oficial da Marinha Mercante (Pata, 2016, p.104-105), contribui para a criação da coleção comparativa com a oferta de vários objetos provenientes de Angola e Brasil (Rocha, 1905, p.81), mas será sobretudo em 1897 que se regista a sua maior doação: 89 objetos provenientes de Moçambique e África do Sul (p.71-89). Numa carta de 1896 dirigida a Santos Rocha, refere a intenção de contribuir para o enriquecimento do museu com um conjunto de objetos que simbolizavam o êxito dos portugueses perante a resistência das comunidades locais: “Venho cumprir a promessa que fiz de mandar mais armas gentílicas”, identificando a proveniência de objetos de Moçambique.^16^

Três anos depois da criação do museu, a seção de comparação contava com 1.084 objetos (Rocha, 1897, p.81-86), sendo os principais doadores Antonio d’Oliveira e Silva Júnior, Bernardo Augusto Lopes, João Francisco Branco, José Marques Pinto e João Maria Simões (p.253), todos naturais da Figueira da Foz ou da região.

João Francisco Branco (1834-1905) foi um oficial da Marinha Mercante (Pata, 2016, p.104-105) que negociava entre a Bahia (Brasil) e a “Costa dos Escravos”, região na África Ocidental que compreendia partes dos atuais Benim, Togo e Nigéria ocidental (Mann, 2007, p.126). Viveu em Ouidah ou Ajudá (Benim) entre 1860 e 1873 (Castillo, 2016, p.40), local de proveniência de alguns dos objetos por ele doados ao museu entre 1894 e 1896. José Marques Pinto (1859-1915) foi um comerciante da Figueira da Foz que ofereceu objetos provenientes do “Congo”, em 1894. João Maria Simões (1860-1906), por sua vez, encaminhou do Brasil “Armas dos índios Parentintins, das margens do rio Madeira, afluente do Amazonas” (Rocha, 1905, p.69), entre 1895 e 1897.

No ano de 1900, os registos de incorporação remetem para a oferta de 200 artefactos pelo militar João dos Santos Pereira Jardim (1865-1907)^17^ – provenientes de Timor (Rocha, 1905, p.90-92, 98-100) e do “Congo português”^18^ (Rocha, 1907b, p.1-3)*.* O seu testemunho pessoal permite-nos aceder ao contexto preciso de recolha de alguns objetos, nesse caso, os obtidos quando comandava uma expedição militar em Angola (Jardim, 1904, p.252). A escultura denominada pelo próprio como Deus da Guerra aparece descrita no Catálogo Geral como “tomado na povoação de Fardiabengo, região de Quincunguila, por ocasião da guerra do Quitamboco, em março de 1902” (Rocha, 1905, p.109), e cujo momento de recolha, durante uma campanha militar, deste e de outros objetos, por João Jardim, podemos encontrar descrita na *Revista Portuguesa Colonial e Marítima*, num texto intitulado “A expedição à Quincunguila na circunscrição do Ambrizette”^19^ (Jardim, 1904, p.29-30).

Se relativamente a João Jardim conseguimos aceder a fontes que nos permitem conhecer o contexto de recolha, para outros atores associados ao colecionismo colonial identificados no MMSR não se encontram referências. Exemplo disso é o caso da doação de um conjunto de objetos provenientes do Brasil, por Pedro Augusto Ferreira (1833-1913). Está documentada a doação de “Lanças de madeira, setas de ponta de bambu e madeira, arcos, rede, zarabatanas e setas respectivas, frasco de curara para as envenenar, bolsa d’algodão para as mesmas, colares de sementes e trajes de penas e de palha ou casca d’arvore” (Rocha, 1905, p.68). Pelo que conseguimos apurar, esse doador, abade de Miragaia, não teve contato direto com o território brasileiro, desconhecendo-se o contexto de aquisição e mobilização dos mencionados objetos desde o Brasil.

A Sala de Comparação, de acordo com a descrição do *Catálogo Geral* (Rocha, 1905, p.67-109), possuía um móvel envidraçado, dividido em seis partes, denominadas “estantes” e ordenadas alfabeticamente. Nelas se exibiam, numa organização geográfica, os artefactos desta seção de comparação (Figura 6). Depois de 1910, data do falecimento do seu fundador, o museu entrou numa fase de letargia que se prolongou até 1945, quando reabriu no edifício dos Paços do Concelho, adaptado para o efeito (In memoriam..., 1945). A coleção de comparação seria reorganizada e os seus artefactos integrados na Sala de Etnografia, na Sala de Cerâmica, na Sala de Escultura Religiosa e na Sala de Curiosidades. Em 1975, o museu foi transferido para um novo edifício, e a Sala de Etnografia – onde persistiam as coleções incorporadas no século XIX e inícios do XX – abriu ao público em 25 de abril de 1981 com uma exposição de objetos de Angola e Moçambique, organizados tematicamente (Pereira, 1982).

**Figura 8:**
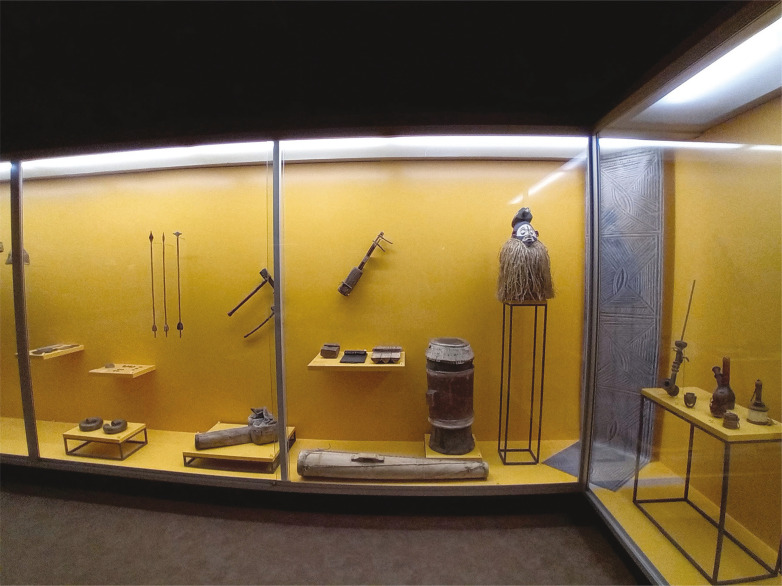
: Sala de Etnografia do Museu Municipal Santos Rocha, 2024 (Fonte: Museu Municipal Santos Rocha)

Na comemoração do centenário do museu, em 1994, a dinâmica expositiva da Sala de Etnografia é ampliada, ocupando uma segunda ala do edifício. Segundo o roteiro publicado em 1995 (Museu..., 1995), a “Coleção africana”, com objetos de Angola e Moçambique, continuaria em exibição, mas passou a integrar também uma seleção de objetos do “Golfo de Benin e [do povo] Yoruba”.^20^ Incluíram-se, pela primeira vez, “Coleções orientais” que abrangem objetos provenientes do Japão, China e Timor. Essa dinâmica altera-se pela última vez em 2014, quando a Sala de Etnografia volta a ocupar o seu espaço original, exibindo objetos provenientes de Angola e Timor. No mesmo ano, o museu criou a reserva visitável, expondo a totalidade da coleção etnográfica e reutilizando o mobiliário do século XIX.

## Considerações finais

Os dados apresentados neste artigo demonstram a diversidade de tópicos e de questões suscitados pela pesquisa de proveniência das coleções não europeias. Em primeiro lugar a ligação entre arqueologia e colonialismo. A criação e o desenvolvimento de coleções de comparação para apoiar o desenvolvimento dos estudos pré-históricos suscitou a criação de redes de atores que angariaram objetos e coleções para representar nos museus europeus o modo de vida dos povos então considerados “selvagens contemporâneos”, uma expressão de época que reflete as perspetivas coloniais sobre as hierarquias do valor humano. A arqueologia reforçava, por meio dessas categorizações, as narrativas sobre a suposta inferioridade dos grupos colonizados.

A investigação desenvolvida sobre as coleções comparativas do Museu Nacional de Arqueologia e do Museu Municipal Santos Rocha evidenciou a predominância de objetos provenientes do continente africano e incorporados numa época de campanhas de ocupação territorial em finais do século XIX e inícios do século XX. Portugal disputava com outras potências europeias a apropriação efetiva dos territórios africanos, e as campanhas militares para dominar a resistência das populações locais foram, como este estudo demonstra, um dos meios para a aquisição de objetos que surgem nas duas instituições. Vários militares e administradores coloniais surgem como doadores nos dois museus. Fontes históricas como os estudos etnográficos e os relatórios realizados por militares tornam evidente a posição de poder desses atores no momento de constituição de coleções.

Identificámos outras ofertas de cortesia às instituições museológicas cujos processos de compra/obtenção ou identidade dos doadores são difíceis de rastrear. Por outro lado, os dados biográficos de parte dos atores registados, como o abade de Miragaia, o farmacêutico César Azevedo Pires ou o próprio José Leite de Vasconcelos, não apresentam evidências de viagens, estadias ou residências no continente africano. É provável que essas coleções tenham sido obtidas por meio de agentes coloniais – militares, administradores, missionários ou comerciantes – que as transportaram até ao continente europeu e as comercializaram e/ou facultaram às suas redes de contatos.

Enquanto no Museu Nacional de Arqueologia são frequentes as doações de militares, no Museu Municipal Santos Rocha destacam-se as doações de agentes comerciais ligados à marinha mercante, como exemplificámos pelos casos de Bernardo Augusto Lopes e de João Francisco Branco. Esses agentes são por vezes convocados pelos próprios diretores de museus a recolher objetos específicos para enriquecer as coleções: exemplo da doação de Bernardo Augusto Lopes. Para esses atores é motivo de orgulho e evidência de civilidade contribuir para o enriquecimento do conhecimento por meio da constituição de coleções únicas, que serão exibidas nos museus nacionais ou regionais. Cooperando com o desenvolvimento dos estudos pré-históricos, os objetos não europeus que integravam as coleções comparativas contribuíam também para espelhar a realidade do colonialismo e evidenciar a diversidade humana. Mais tarde, com o Estado Novo (1933-1974), e consolidados os estudos pré-históricos, essas coleções adquirem nova vocação e designação – Sala de Etnografia no MMSR e Sala de Etnografia Ultramarina no MNA –, assumindo-se como instrumento de propaganda do Império Colonial Português. Após o 25 de Abril de 1974, e consequente democratização de Portugal, a coleção etnográfica do Museu Nacional de Arqueologia perde protagonismo em favor da especialização arqueológica da instituição. Todas as coleções etnográficas (europeias e não europeias) são colocadas em reserva. No Museu da Figueira da Foz, a coleção etnográfica permanece em exposição, assumindo, como referimos, várias disposições até à atualidade.

A investigação desenvolvida no âmbito do projeto TRANSMAT mostra como as coleções etnográficas não europeias do MNA e MMSR constituem arquivos da história colonial. Essas coleções, quando combinadas com outras fontes históricas e publicações da época, contribuem para uma compreensão mais completa do imperialismo e dos seus efeitos nas colónias e na metrópole. Identificando momentos históricos específicos, a pesquisa de proveniência baseada em fontes históricas heterogéneas pode não só contribuir para a criação de renovadas perspetivas históricas, mas também para redefinir as possibilidades de exposição dos objetos e das coleções (Deußen, Karakis, 2022, p.5). Na documentação produzida nos museus para administrar e expor os artefactos encontramos uma linguagem que reflete ações, atitudes e interpretações coloniais. Essa informação foi sendo replicada ao longo dos anos, pelos sucessivos serviços de inventários e curadores que continuam a utilizar as mesmas linguagem, categorias e designações convencionadas pelos primeiros diretores e pelos próprios doadores.

O que apresentamos neste artigo constitui apenas uma amostra do potencial dos dados recolhidos no âmbito deste projeto de investigação e da reflexão que se deve promover sobre esses acervos da histórica colonial. Muitas vias de pesquisa ficaram abertas e, no futuro, serão desenvolvidas. Um futuro que deverá não apenas alargar investigações similares para outros museus portugueses, como incorporar metodologias transdisciplinares de investigação, que, consequentemente, envolverão os descendentes das comunidades de origem dos objetos e a sua diáspora.

## NOTAS

2 Bertrand Daugeron (2009) estudou o “Musée des Antiques” da Biblioteca Nacional em Paris e a dispersão das coleções etnográficas que integravam o projeto de comparação que lhe estava associado. Destaca-se igualmente o trabalho de Christine Lorre (2015, 2024) sobre as perspetivas comparatistas no Musée d’Archéologie Nationale de Saint-Germain-en-Laye, o estudo de Adèle Chevalier (2020) sobre essa tipologia de coleções em três instituições museológicas europeias e o estudo de Beatrice Falcucci (2022) sobre os objetos etnográficos de contextos coloniais africanos durante o final do século XIX e o início do século XX, em Itália.

3 O período entre 1893 e 1910 corresponde ao início de construção da coleção (um ano antes da inauguração em 1894) e ao ano de falecimento do fundador do museu.

4 Constrangimentos vários impediram o acesso à coleção no Museu Nacional de Arqueologia.

5 Fundado como Museu Etnográfico Português, o atual Museu Nacional de Arqueologia modificaria em 1897 a sua designação para Museu Etnológico Português.

6 As seções do museu eram na época as seguintes: I. Antiguidades nacionais; II. Etnografia portuguesa; III. Seção antropológica; IV. Seção comparativa; V. Objetos indeterminados; VI. Biblioteca do Museu (Vasconcelos, 1915, p.170).

7 A revista continua a ser publicada até à atualidade, mas a partir de 1920 deixou de incluir os registos de “aquisição”.

8 A publicação, criada dois anos depois da fundação do museu, em 1895, registou as aquisições efetuadas desde 1893.

9 Não contabilizamos nesta análise a aquisição de documentos e livros provenientes do estrangeiro.

10 De salientar igualmente que foram registadas doações sem mencionar o doador associado, bem como o fato de parte dos registos não serem precisos quanto à forma de incorporação ou de proveniência.

11
*Livro de registo de matrículas dos sócios da Sociedade Farmacêutica Lusitana*; PT/OF/CDF/C-A/004-001/0007. Disponível em: http://www.cdf.pt/archeevo/viewer?id=1001071&FileID=168848. Acesso em: 3 nov. 2023.

12 Na sequência da viagem que o seu diretor efetuou ao Egito, em 1909, uma coleção de mais de duas dezenas de objetos entraram no museu (Carvalhaes, 1911, p, p.120-121).

13 Veja-se, por exemplo, o conjunto de objetos adquirido por José Leite de Vasconcelos em 1906: “setenta armas dos indigenas da África portuguesa: azagaias, lanças, punhais, espadas, travesseiros e um tambor” (Campos, 1907, p, p.107). De diversas proveniências também obteve Vasconcelos, por compra, o seguinte: “Objetos da África portuguesa, fabricados em madeira: uma grande caneca com asa; um travesseiro; duas banquinhas, do Bailundo” (p.111). O Bailundo, ou Mbailundu, foi entre 1891 e 1903, de forma sucessiva, intervencionado militarmente, ocorrendo em 1902-1903 a denominada Segunda Guerra Luso-Ovibundo (Wheeler, Christensen, 1973).

14 A equipa do museu não identificou no acervo fotográfico do MNA fotografias da coleção em análise anteriores à década de 1960.

15 Arquivo Histórico da Câmara Municipal da Figueira da Foz. Livro de Inumação nº 4, 1898. Registo de óbitos do cemitério septentrional. Nº de ordem 364, p.37v. e 38.

16 Arquivo do Museu Municipal Santos Rocha.

17 Arquivo Distrital de Coimbra. Registo de Batismos da Paróquia de Santa Cruz, 1865. N.º de ordem 91, p.28v. e 29.

18 Refere-se ao Protetorado do Congo Português, estabelecido em 1885.

19 População situada na costa atlântica, a norte de Luanda, Angola.

20 Uma região localizada na costa da África Ocidental, abrangendo áreas dos atuais Benim, Togo e Nigéria.
